# The Minor Wall-Networks between Monolignols and Interlinked-Phenolics Predominantly Affect Biomass Enzymatic Digestibility in *Miscanthus*

**DOI:** 10.1371/journal.pone.0105115

**Published:** 2014-08-18

**Authors:** Zhengru Li, Chunqiao Zhao, Yi Zha, Can Wan, Shengli Si, Fei Liu, Rui Zhang, Fengcheng Li, Bin Yu, Zili Yi, Ning Xu, Liangcai Peng, Qing Li

**Affiliations:** 1 National Key Laboratory of Crop Genetic Improvement, Biomass and Bioenergy Research Centre, College of Plant Science and Technology, Huazhong Agricultural University, Wuhan, China; 2 National Key Laboratory of Crop Genetic Improvement, Biomass and Bioenergy Research Centre, College of Life Science and Technology, Huazhong Agricultural University, Wuhan, China; 3 Department of Biotechnology, Hunan Agricultural University, Changsha, China; 4 Jiangsu Key Laboratory for Biomass-based Energy and Enzyme Technology, Huaiyin Normal University, Huaian, China; 5 National Key Laboratory of Crop Genetic Improvement, Biomass and Bioenergy Research Centre, College of Science, Huazhong Agricultural University, Wuhan, China; Iowa State University, United States of America

## Abstract

Plant lignin is one of the major wall components that greatly contribute to biomass recalcitrance for biofuel production. In this study, total 79 representative *Miscanthus* germplasms were determined with wide biomass digestibility and diverse monolignol composition. Integrative analyses indicated that three major monolignols (S, G, H) and S/G ratio could account for lignin negative influence on biomass digestibility upon NaOH and H_2_SO_4_ pretreatments. Notably, the biomass enzymatic digestions were predominately affected by the non-KOH-extractable lignin and interlinked-phenolics, other than the KOH-extractable ones that cover 80% of total lignin. Furthermore, a positive correlation was found between the monolignols and phenolics at *p*<0.05 level in the non-KOH-extractable only, suggesting their tight association to form the minor wall-networks against cellulases accessibility. The results indicated that the non-KOH-extractable lignin-complex should be the target either for cost-effective biomass pretreatments or for relatively simply genetic modification of plant cell walls in *Miscanthus*.

## Introduction

Lignocellulose is the most abundant and sustainable biomass on the earth for biofuels and other chemical products [1]–[3]. The current biomass process mainly involves three major steps, namely, physical and chemical pretreatment for plant cell wall destruction, enzymatic hydrolysis for saccharification, as well as yeast fermentation for ethanol production [4]. However, biofuel production remains extremely costly due to biomass recalcitrance, and could result in secondary environmental pollution [5]. Generally, recalcitrance is determined by the wall polymer components and their various interactions [6]–[9]. For recalcitrance reduction, the genetic modification of plant cell walls is considered a promising solution in bioenergy crops and it has become essential in understanding the effects of wall polymer on biomass digestibility [10]–[12].

Plant cell walls are mainly composed of cellulose, hemicelluloses, and lignin with relatively small amounts of pectin and proteins [13], [14]. In biomass hydrolysis, cellulose crystallinity is reportedly a negative parameter [15], [16], whereas hemicelluloses are a positive and dominant factor [12]. Lignin is an amorphous polymer with phenylpropane units, which mostly comprise three monomers, namely, p-hydroxyphenyl (H), guaiacyl (G), and syringyl (S) [17]. These monomers are linked by ether-, ester- and C-C bonds that are irregularly repeated [18], [19]. Lignin has been associated with biomass recalcitrance, and the biomass saccharification rate is strongly affected by the level and the monomer composition of lignin [16], [20]. However, the phenolic acid-based interconnection among lignin and wall polysaccharides has not yet been fully understood [21]. Despite that the lignin level and the S/G ratio show the negative effects on biomass saccharification in transgenic switchgrass [22], [23], the higher S/G ratio in natural populus was recently reported to exhibit a positive effect on sugar release while the negative effect of lignin level is less pronounced [20].

*Miscanthus* is a C4 perennial grass with an extremely high biomass yield, and is currently considered as the leading candidate for biofuel feedstocks. With its origin in East Asia, we collected over 1400 natural *Miscanthus* accessions with a rich and stable germplasm resource [12], [24]. In this study, we initially selected 79 representative *Miscanthus* natural accessions with a diverse cell wall composition and structure [24], [25]. An integrative analysis was performed among lignin level/composition, interlinked-phenolics and biomass digestibility of the selected *Miscanthus* samples. The mechanism was then interpreted about the lignin negative effect on biomass enzymatic saccharification under NaOH and H_2_SO_4_ pretreatments at three concentrations.

## Results and Discussion

### Diversity of lignin level and monomer composition in *Miscanthus*

**Figure 1:**
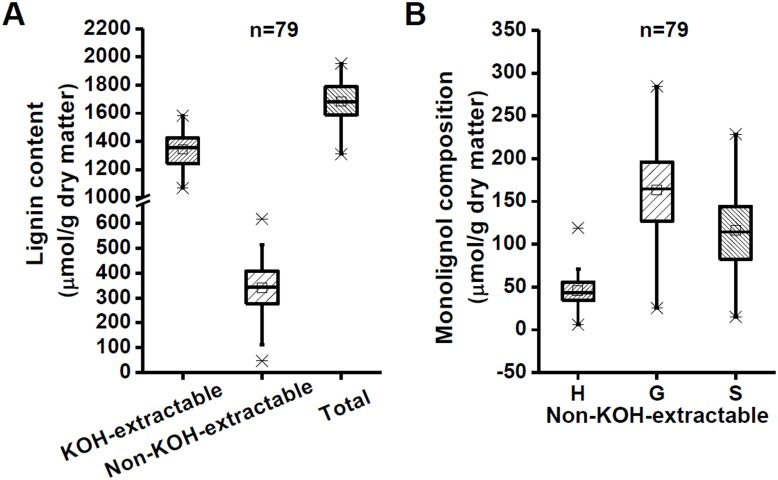
Variation of lignin content and monolignol composition in *Miscanthus* (n = 79). (A) Lignin content; (B) Monolignol composition.

Diverse lignin contents and monomer compositions were determined in 79 representative *Miscanthus* germplasm accessions (Fig. 1). The KOH-extractable and non-KOH-extractable lignin levels of *Miscanthus* accessions ranged from 831.47 µmol/g to 1582.40 µmol/g and from 46.35 µmol/g to 618.25 µmol/g, respectively (Table S1; Fig. 1A). In general, *Miscanthus* displayed a diverse lignin monomer composition. For example, an average of 48.63% G was much higher than the other two monomers, H at 24.16% and S at 27.21%. Notably, despite the non-KOH-extractable residue covered only 20% of total lignin (Table S1), it also showed a high variation in each monomer, i.e., H, 6.00 µmol/g to 119.18 µmol/g; G, 25.27 µmol/g to 284.39 µmol/g; and S, 15.08 µmol/g to 228.81 µmol/g (Fig. 1B). The diversity of lignin levels and monolignol compositions, particularly in the non-KOH-extractable form, suggested that lignin may play a distinct role in biomass enzymatic digestibility of the selected *Miscanthus* samples.

### Variation of interlinked-phenolics in *Miscanthus*

**Figure 2:**
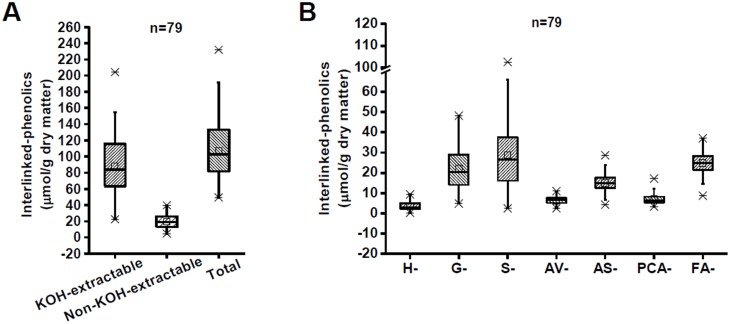
Variation of interlinked-phenolics in *Miscanthus* (n = 79). (A) Interlinked-phenolics in the KOH-extractable and non-KOH-extractable residues; (B) Total interlinked-phenolic compositions. H-: *p*-Hydroxybenzaldehyde, G-: Vanillin, S-: Syringaldehyde, AV-: Acetovanillone, AS-: Acetosyringone, PCA-: *p-*Coumaric acid, FA-: Ferulic acid, SA-: Sinapic acid.

Lignin interacts with other wall polymers through the interlinked-phenolics [26], and thus, we detected seven major ester- and ether-linked phenolic compounds in the *Miscanthus* (Fig. 2). The selected 79 *Miscanthus* samples exhibited the KOH-extractable phenolics that ranged from 22.47 µmol/g to 204.35 µmol/g, whereas the non-KOH-extractable phenolics ranged from 4.85 µmol/g to 39.82 µmol/g (Fig. 2A). Likewise, the non-KOH-extractable biomass residue also contained only 20% of total interlinked-phenolics. Among the seven interlinked-phenolics that were examined, *Miscanthus* samples showed a relatively high variation in syringaldehyde (S-), vanillin (G-), acetosyringone (AS-), and ferulic acid (FA-) (Fig. 2B). Hence, the selected *Miscanthus* accessions indicated diversity in monolignol and interlinked phenolic composition.

### Lignin effect on biomass saccharification

Considering the selected *Miscanthus* samples that exhibited diversity in the monolignol and interlinked phenolic compositions, their biomass enzymatic digestions were detected after various chemical pretreatments. The biomass digestibility (saccharification) was defined by Huang et al. [24] by accounting for the hexoses yield (hexoses/cellulose) released from the enzymatic hydrolysis of a crude cellulase mixture of lignocellulose upon pretreatment. After pretreated with NaOH (0.5%, 1%, and 4%) and H_2_SO_4_ (0.25%, 1%, and 4%), the selected *Miscanthus* accessions exhibited a high variation in biomass digestibility, as hexoses yield. As a result, a few *Miscanthus* accessions could be considered as the potential bioenergy crops.

**Figure 3:**
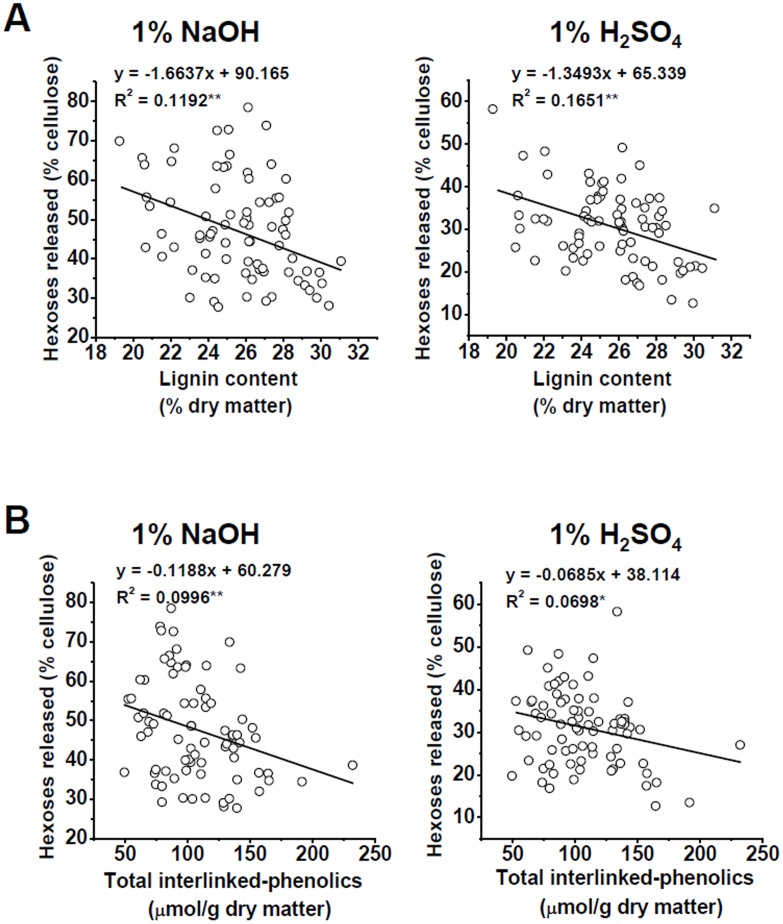
Correlation analysis among lignin, interlinked-phenolics and biomass saccharification in *Miscanthus*. (A) Correlation between lignin content and hexoses yield; (B) Correlation between total interlinked-phenolics and hexoses yield. * and ** Indicated the significant correlation coefficient values at *p*<0.05 and 0.01 (n = 79), respectively.

With regard to the selected *Miscanthus* samples, a correlation analysis was performed among lignin, interlinked-phenolics, and biomass saccharification after pretreatments with NaOH and H_2_SO_4_ at three concentrations (Figs. 3 and S1). Among the 79 *Miscanthus* accessions, the total lignin level and interlinked-phenolics negatively affected the hexoses yield after the pretreatment with various concentrations of NaOH (0.5%, 1%) and H_2_SO_4_ (0.25%, 1%) (Figs. 3 and S1A). Only lignin exhibited a negative effect after a 4% NaOH or 4% H_2_SO_4_ pretreatment (Fig. S1B). Despite the relatively low correlation (*R* ^2^) values, the correlation coefficients reached significant levels at *p*<0.01 or *p*<0.05 (n = 79). Hence, lignin could exhibit the negative influence on biomass digestibility in *Miscanthus*. Although previous study has reported that the lignin level negatively affect biomass digestibility in *Miscanthus* [12], the characterization of diverse *Miscanthus* germplasm accessions in the current study could account for the mechanism of the negative effects of lignin on biomass saccharification as described below.

### Monolignol and interlinked-phenolics impact on biomass digestibility

**Figure 4:**
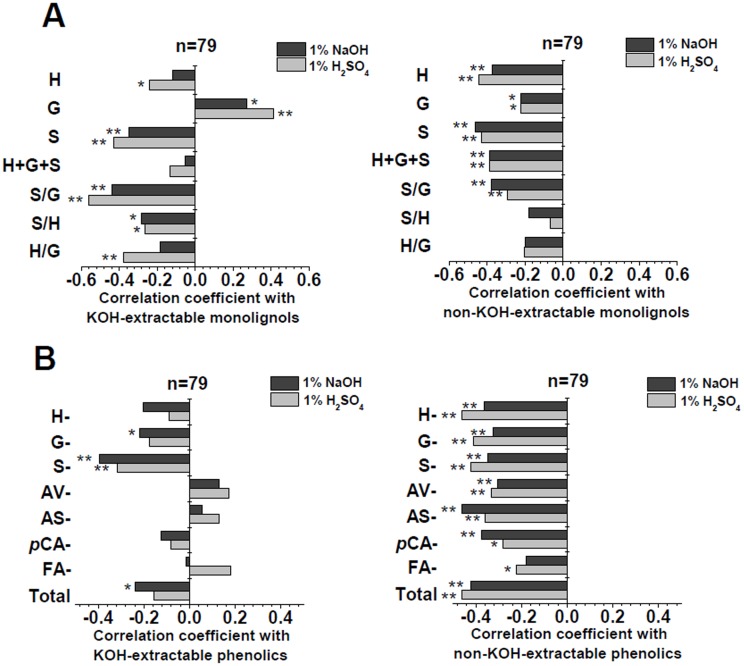
Correlation analysis among monolignols, seven interlinked-phenolics and biomass saccharification in *Miscanthus*. (A) The KOH-extractable and non-KOH-extractable monolignols; (B) The KOH-extractable and non-KOH-extractable interlinked-phenolics. * and ** Indicated the significant correlation coefficient values at *p*<0.05 and 0.01 (n = 79), respectively.

To gain insights on the negative effects of lignin on biomass digestions, we conducted a correlation analysis between three major monolignols and interlinked-phenolics in the KOH-extractable and non-KOH-extractable biomass residues of *Miscanthus* samples (Figs. 4, S2 and S3). In the KOH-extractable residue of *Miscanthus* accessions, S and S/G ratio negatively affected the biomass digestibility at *p*<0.01 or 0.05 levels (n = 79); however, the total monomers (H+G+S) produced no significant effect (Figs. 4A, S2A, and S3A). By contrast, S, S/G and the total monomers in the non-KOH-extractable exhibited a significantly negative effect on the hexoses yields after pretreated with various concentrations of NaOH (0.5% and 1%) and H_2_SO_4_ (0.25%, 1%, and 4%) (Figs. 4A, S2B, and S3B). In addition, despite H monomer in the non-KOH-extractable residue also exhibited high negative coefficient values, the S/H and H/G ratios did not show any significant correlations (*p*>0.05). Thus, the results indicated that the S level or S/G ratio (other than S/H and H/G ratios) in the non-KOH-extractable, and not in the KOH-extractable residue, could account for the negative effect of lignin on biomass digestibility in *Miscanthus*. Notably, the seven major interlinked-phenolics in the non-KOH-extractable residue also exhibited a significantly negative effect (*p*<0.01 or *p*<0.05) on biomass enzymatic digestibility after pretreated with three concentrations of NaOH and H_2_SO_4_, except for FA- (Figs. 4B, S2D, and S3D). In addition, compared with the KOH-extractable residues, the non-KOH-extractable residues contain much more S-monolignol and interlinked-S and AS compounds with less Ara and FA, suggesting that the non-KOH-extractable residues should present the S and AS-rich wall polymer networks.

Nevertheless, the most interlinked-phenolics in the KOH-extractable residue were not correlated with biomass saccharification (Figs. 4B, S2C, and S3C). Hence, despite that the non-KOH-extractable biomass residue contained only 20% of the total lignin in *Miscanthus* samples (Table S1), their monolignols and interlinked-phenolics could predominately determine the negative effect of total lignin on biomass digestibility under various pretreatments in *Miscanthus* accessions. It was consistent with the previous report that the non-KOH-extractable biomass residue was significantly correlated with the lignocellulose residues released from various physical and chemical pretreatments in terms of their biomass enzymatic digestibility [25]. The results also confirmed that biomass enzymatic saccharification could be fundamentally determined by plant cell wall structures [25], [27].

To our knowledge, it was first time to report about the non-KOH-extractable lignin distinct effect on biomass enzymatic saccharification in plants. This finding indicated that the non-KOH-extractable lignin should be the target either for genetic modification of plant cell walls or for chemical and physical pretreatments of biomass. Notably, although three monomers (S, G, H) of the non-KOH-extractable lignin were all negatively correlated with biomass digestibility, only S/G ratio, other than S/H or H/G, displayed a significant effect (Fig. 4A). This result suggested that reducing S proportion in the non-KOH-extractable lignin should be the priority for lignin modification in *Miscanthus*. However, three monomers of lignin and their ratios have been determined with dual effects on biomass enzymatic hydrolysis in rice, wheat and sweet sorghum [16], [28], suggesting that the specific lignin modification may depend on the plant species.

### Mechanism on lignin negative effect on lignocellulose enzymatic hydrolysis

**Figure 5:**
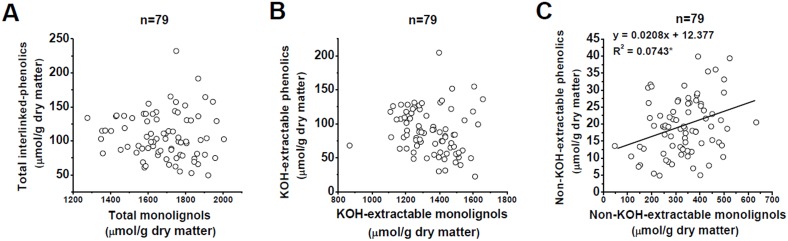
Correlation analysis between monolignols and interlinked-phenolics in *Miscanthus*. (A) Total monolignols and interlinked-phenolics; (B) The KOH-extractable monolignols and interlinked-phenolics; (C) The non-KOH-extractable monolignols and interlinked-phenolics. * Indicated the significant correlation coefficient value at *p*<0.05, (n = 79).

To further understand lignin negative impact on biomass digestibility, we performed a correlation analysis between three monolignols and interlinked-phenolics in the two types of biomass residues among total 79 *Miscanthus* accessions (Figs. 5 and S4). In general, no significant correlation was observed among the total monolignols and interlinked-phenolics in *Miscanthus* (Fig. 5A). However, *Miscanthus* accessions exhibited a positive correlation at *p*<0.05 level in the non-KOH- extractable, rather than in the KOH-extractable residue (Figs. 5B and 5C). Notably, seven major ester- and ether-interlinked-phenolics were positively correlated with the total lignin only in the non-KOH-extractable residues of *Miscanthus* at *p*<0.01 or 0.05 levels (Fig. S4B). Despite that the ester-interlinked-phenolics could be effectively extracted with alkali chemicals, they remained at minor levels in the non-KOH-extractable residues from 22.4% KOH extraction (data not shown), suggesting that the ester-interlinked-phenolics should also play a role in wall polymer network formation. Because the wall-networks formed by ester- and ether-interlinked-phenolics among lignin and other wall polymers may act as the barrier hindering the enzyme penetration into the cellulose surface [29], the significantly positive correlation between the non-KOH-extractable monolignols and interlinked-phenolics suggested their association to establish wall-networks against enzymatic accessibility. It also indicated that the pretreatments (0.5%, 1%, and 4% NaOH; 0.25%, 1%, and 4% H_2_SO_4_) used in this study could not remove the non-KOH-extractable lignin and interlinked-phenolics, due to the KOH extraction concentration at 22.4%. Nevertheless, while 8% NaOH or 8% H_2_SO_4_ was even used as one-step pretreatment, we found that the biomass enzymatic digestibility was not much increased in the *Miscanthus* samples examined in this study. It suggested that two-step pretreatments with alkali and acid or other integrated approaches may be attempted in order for removal of the non-KOH-extractable lignin and interlinked-phenolics.

In addition, despite the KOH-extractable residue contained almost 80% of total lignin (Fig. 1), the non-significant correlation between the KOH-extractable lignin and interlinked-phenolics suggested that most lignin could not well establish the linkages with the phenolics to form the complete wall-networks in *Miscanthus*. Hence, although the S/G ratio in the KOH-extractable residue displayed a significantly negative correlation with the lignocellulose enzymatic hydrolysis (Fig. 4A), it should not determine the lignin negative impact on biomass digestibility in *Miscanthus*. In other words, since the non-KOH-extractable biomass residues maintain 20% total hemicelluloses in *Miscanthus* [25], we assumed that the non-KOH-extractable hemicelluloses may strongly interact with lignin via interlinked phenolics. Hence, the minor non-KOH-extractable biomass residue should predominately affect lignocellulose enzymatic hydrolysis by its monolignols and interlinked-phenolics forming the tight wall-networks with hemicelluloses against cellulase accession in *Miscanthus*.

### Potential cell wall modification for high biomass digestibility

**Figure 6:**
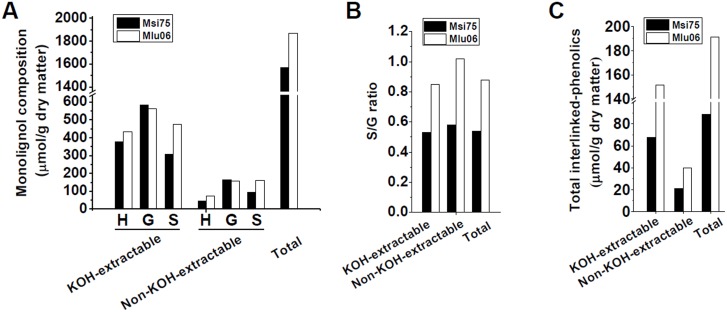
Analysis of monolignol and interlinked-phenolics compositions in the representative *Miscanthus* samples. (A) Monolignol composition; (B) S/G ratio; (C) Total interlinked-phenolics.

Based on the previous analysis, one pair of representative samples were selected from the *Miscanthus* accessions (Figs. 6 and S5). The *Miscanthus* accession (Msi75) with a relatively higher biomass digestibility exhibited lower lignin level, interlinked phenolic composition, and S/G ratio compared with those of the paired sample (Mlu06) (Fig. 6). The results confirmed that lignin and interlinked-phenolics exhibited the negative effects on biomass digestibility in *Miscanthus*. Furthermore, *Miscanthus* accession (Msi75) exhibited an increased biomass enzymatic digestibility at a similar rate compared with the paired sample (Mlu06) after pretreatment with three concentrations of NaOH and H_2_SO_4_ (Fig. S5A), consistent with the previous findings about both hexoses yields (% cellulose) and total sugars yields in *Miscanthus* [25]. Using scanning electron microscopy, we also observed rougher biomass surface on the Msi75 sample compared with its paired sample (Mlu06) after NaOH and H_2_SO_4_ pretreatments and sequential enzymatic hydrolysis (Fig. S5B). The rough surface samples may enhance the cellulase compatibility and accessibility for high enzymatic activity as discussed by Xu et al (2012).

*Miscanthus* is a typical C4 plant with enormous biomass residues for biofuels. However, as plant biomass contains many different cell types with diverse wall components, it remains hard to identify desirable cell walls for high biomass digestibility [11]. Due to diversity of cell wall biological functions, any genetic modification of plant cell walls could consequently cause plant growth defect and mechanic strength reduction. In this work, however, we have identified several natural *Miscanthus* accessions with normal plant growth and high biomass digestibility from large collections of *Miscanthus* germplasms such as the Msi75 sample. This is because *Miscanthus* with self-incompatibility at flowering, can evolve into different species with a diverse germplasm that is suitable for plant growth under various environmental conditions [11], [21], [30]. Thus, the selected *Miscanthus* natural germplasm accessions should be directly used as desire bioenergy crops for biofuel purposes.

Notably, as the non-KOH-extractable residue only covers about 20% of total lignin, its genetic modification should cause relatively less defects on plant growth and development than that of the KOH-extractable one, suggesting that reducing of the non-KOH-extractable lignin complex could be the relatively simply way for genetic modification of plant cell walls in bioenergy crops. More importantly, as all *Miscanthus* accessions exhibited extremely lower interlinked-phenolics than that of lignin in the non-KOH-extractable residues (Figs. 1A and 2A), removal of the non-KOH-extractable interlinked-phenolics should be a cost-effective pretreatment approach for high biomass enzymatic digestibility.

On the other hand, replacing the non-KOH-extractable interlinked-phenolics with other soluble substances such as proteins, may be the way for satisfying with the needs of both plant normal growth under various environmental stresses, and high biomass enzymatic digestibility upon different pretreatments in *Miscanthus*.

## Conclusions

Total 79 representative *Miscanthus* samples have been determined with a variation of lignocellulose enzymatic digestibility and a diversity of three monolignols (S, G, H) and seven interlinked-phenolics compositions. Correlative analysis has indicated that either monolignols (S/G ratio) or interlinked-phenolics could negatively affect biomass enzymatic digestibility under NaOH and H_2_SO_4_ pretreatments. Integrative analysis has also suggested that the minor non-KOH-extractable monolignols and interlinked-phenolics should have a coordination to form the wall-network that could predominately determine lignin negative influence on lignocellulose saccharification. The findings could provide the potential approaches for simply genetic modification of plant cell walls in *Miscanthus*, as well as for cost-effective physical and chemical pretreatments toward high biomass digestibility.

## Materials and Methods

### Plant materials

*Miscanthus* samples were selected from wild *Miscanthus* germplasm resources collected nation-wide in China. The mature stem tissues of *Miscanthus* samples were supplied by the laboratory of Dr. Zili Yi at Hunan Agricultural University, Changsha, China[12], [24]. The collected samples of mature stem tissues were inactivated at 105 °C for 10 min and dried at 50 °C until with constant weight. The dried tissues were ground through a 40 mesh screen, and stored in a dry container until use. All samples were collected from 5–10 individual mature stem tissues and the ground powders were mixed well prior to use for cell wall composition analysis, biomass pretreatments and enzymatic hydrolysis. No specific permission was required for the field study, which was carried out in the specific experimental field for energy plants. In addition, *Miscanthus* is not listed as the endangered or protected species in China.

### Plant cell wall fractionation

The procedures of plant cell wall fractionation were described by Peng et al. [31] with minor modification by Li et al. [25]. The extracted crude cell wall residues were extracted with 0.5% (w/v) ammonium oxalate for 1 h in a boiling water bath. The remaining pellet was extracted with 4 M KOH containing 1.0 mg mL^−1^ sodium borohydride for 1 h at 25 °C, and the combined supernatant was neutralized, dialyzed and lyophilized as KOH-extractable fractionations. The remaining pellet contained the non-KOH-extractable fractionations. All samples were carried out in biological triplicate for wall fractionations.

### Colorimetric assay for total hexoses and pentoses

UV/VIS Spectrometer (Shanghai MAPADA Instruments Co., Ltd. V-1100D) was applied for total hexoses and pentoses assay. The anthrone/H_2_SO_4_ method was used for total hexoses assay [32]. The orcinol/HCl method was applied for total pentoses assay [33].

### Total lignin determination

Total lignin determination of crude cell wall residues or non-KOH-extractable residues was performed by two-step acid hydrolysis method according to Laboratory Analytical Procedure of the National Renewable Energy Laboratory. The details of the two-type of lignin assay were described by Xu et al. [12].

### Three monolignols (S, G, H) determination by HPLC

The methods were described by Xu et al. [12] with minor modification. Standard chemicals: *p*-Hydroxybenzaldehyde(H), vanillin(G) and syringaldehyde (S) (Sinopharm Chemical Reagent Co., Ltd.) were used for three monolignol proportion determination by HPLC. Three monolignol levels were respectively calculated based on their percentages of total (%) detected by HPLC and total lignin levels determined by the two-step acid hydrolysis method described above. The lignin level of the KOH-extractable or the non-KOH-extractable residues was subjective to the sum up of three monolignols.

The crude cell wall residues or the non-KOH-extractable residues were extracted with benzene-ethanol (2∶1, v/v) in a Soxhlet for 4 h, and the remaining pellet (0.05 g) was added with 5 mL 2 M NaOH and 0.5 mL nitrobenzene in the Teflon gasket with a stainless steel bomb. The bomb was sealed tightly and heated at 170 °C (oil bath) for 3.5 h and stirred at 20 rpm. The chromatographic internal standard (ethyl vanillin) was added to the oxidation mixture. The alkaline oxidation mixture was washed 3 times with 30 mL CH_2_Cl_2_/ethyl acetate mixture (1/1, v/v) to remove nitrobenzene and its reduction by-products. The alkaline solution was acidified to pH 3.0–4.0 with 6 M HCl, and extracted with CH_2_CI_2_/ethyl acetate (3×30 mL) to obtain the lignin oxidation products in the organic phase. The organic extracts were evaporated to dryness under reduced pressure 40 °C, and the oxidation products were dissolved in 10 mL chromatographic pure methanol. Hence, three monolignols of the KOH-extractable residue were calculated based on the subtraction between the crude cell wall residues and the non-KOH-extractable residues.

HPLC analysis: 20 µL solution was injected into HPLC (Waters 1525 HPLC) column Kromat Universil C18 (4.6 mm×250 mm, 5 µm) operating at 28 °C with CH_3_OH:H_2_O:HAc (25∶74∶1, v/v/v) carrier liquid (flow rate: 1.1 mL/min). Calibration curves of all analytes routinely yielded correlation coefficients 0.999 or better, and the detection of the compounds was carried out with a UV-detector at 280 nm.

### Interlinked-phenolics determination by HPLC

The protocols were described by Xu et al. [12] with minor modification. Standard chemicals included *p*-Hydroxybenzaldehyde(H), vanillin(G) and syringaldehyde (S) (from Sinopharm Chemical Reagent Co., Ltd.), Acetovanillone (AV) and acetosyringone (AS) (from Biosharp Co., Ltd.), *trans*-*p*-CA (*p*CA) and *trans*-FA (FA) (purchased from Sigma-Aldrich Co. LLC.).

The crude cell wall residues (0.05 g) were added with 10.0 mL 4 M NaOH containing 1.0 mg/mL NaHSO_3_ and stirred at 170 °C for 2 h in a 25 mL Teflon gasket sealed in a stainless steel bomb at 20 rpm. After transferred to a triangular flask, the lysate was acidified to pH 2.0 with 6 M HCl, the acidified solution was extracted with chloroform (3×10.0 mL), and the combined organic extracts were evaporated to dryness under reduced pressure at 40 °C. The extracts were re-dissolved with 2.0 mL elution phase, filtered by 0.22 µm membrane for total phenolics analysis.

### Chemical pretreatment

The procedures were described by Huang et al. [24] and Zhang et al. [34] with minor modification. H_2_SO_4_ pretreatment: The biomass samples (0.5 g) were added with 10 mL H_2_SO_4_ at three concentrations (0.25%, 1%, 4%, v/v), respectively. The tube was sealed and heated at 121 °C for 20 min in autoclave (15 psi). The sample was shaken at 150 rpm for 2 h at 50 °C, and centrifuged at 3,000 *g* for 5 min. The remaining pellet was washed three times with 10 mL distilled water for enzymatic hydrolysis. The samples added with 10 mL distilled water were shaken for 2 h at 50 °C as the control, and all samples were carried out in biological triplicate.

NaOH pretreatment: The biomass sample (0.5 g) was treated with 10 mL NaOH at three concentrations (0.5%, 1%, 4%, w/v). The sample was shaken at 150 rpm for 2 h at 50 °C, and centrifuged at 3,000 *g* for 5 min. The pellet was washed three times with 10 mL distilled water for enzymatic hydrolysis. The samples with 10 mL distilled water were shaken for 2 h at 50 °C as the control, and all samples were carried out in biological triplicate.

### Enzymatic hydrolysis

The methods were described by Huang et al. [24] and Li et al. [25] with minor modification. The remaining residues from pretreatments were washed 2 times with 10 mL distilled water, and once with 10 mL mixed-cellulases reaction buffer (0.2 M acetic acid-sodium acetate, pH 4.8). The residue sample was added with 10 mL (2 g/L) mixed-cellulases (containing β-glucanase≥6×10^4^ U) and cellulase≥600 U and xylanase≥10×10^4^ U from Imperial Jade Bio-technology Co., Ltd). During the enzymatic hydrolysis, the samples were shaken under 150 rpm at 50 °C for 48 h. After centrifugation at 3,000 *g* for 10 min, the supernatants were obtained for determining amounts of pentoses and hexoses released from enzymatic hydrolysis. The samples with 10 mL reaction buffer were shaken for 48 h at 50 °C as the control, and all samples were carried out in biological triplicate.

### SEM observation

The observation was described by Li et al. [25]. The biomass powder residues were used for SEM observation after NaOH or H_2_SO_4_ pretreatment and enzymatic hydrolysis. The samples were washed with distill water, dried under air, and sputter-coated with gold in a JFC-1600 ion sputter (Mito City, Japan). The surface morphology of the treated samples was sputter-coated with gold and viewed by SEM (JSM-6390/LV, Hitachi, Tokyo, Japan).

### Statistical analysis

Superior Performance Software Systems software package (SPSS 17.0, Inc., Chicago, IL) was applied for the related statistical analyses. Correlation analysis was performed using Spearman's rank correlation analysis at the two-sided 0.05 level of significance (*p <0.05, **p<0.01). The variation and regression analysis are developed using Origin 8.0 software (Microcal Software, Northampton, MA) for the best fit curve from the experimental data.

## Supporting Information

Figure S1 **Correlation of lignin and interlinked-phenolics with hexoses yield released from enzymatic hydrolysis.** (A) Pretreated with 0.5% NaOH and 0.25% H_2_SO_4_; (B) Pretreated with 4% NaOH and 4% H_2_SO_4_ in *Miscanthus* accessions (n = 79). * and ** Indicated the significant correlation coefficient values at *p*<0.05 and 0.01, respectively.
(TIF)

Figure S2 **Correlation of monolignols and interlinked-phenolics with hexoses yield released from enzymatic hydrolysis under various pretreatments.** (A) KOH-extractable monolignols; (B) Non-KOH-extractable monolignols; (C) KOH-extractable phenolics; (D) Non-KOH-extractable phenolics. * and ** Indicated the significant correlation coefficient values at *p*<0.05 and 0.01, respectively (n  = 79).
(TIF)

Figure S3 **Correlation of monolignols and interlinked-phenolics with hexoses yield released from enzymatic hydrolysis under various pretreatments.** (A) KOH-extractable monolignols; (B) Non-KOH-extractable monolignols; (C) KOH-extractable phenolics; (D) Non-KOH-extractable phenolics. * and ** Indicated the significant correlation coefficient values at *p*<0.05 and 0.01, respectively (n = 79).
(TIF)

Figure S4 **Correlation between seven interlinked-phenolics and lignin in** ***Miscanthus*** **.** (A) KOH-extractable lignin; (B) Non-KOH-extractable lignin. * and ** Indicated the significant correlation coefficient values at *p*<0.05 and 0.01, respectively (n = 79).
(TIF)

Figure S5 **Biomass enzymatic digestibility and scanning electron microscopic observation in representative** ***Miscanthus*** **accessions.** (A) Hexoses yields (% cellulose) released from enzymatic hydrolysis after pretreatments of NaOH and H_2_SO_4_ at three concentrations as means±SD (n = 3); (B) SEM imagines of the biomass residues obtained from pretreatments of 1% NaOH and 1% H_2_SO_4_ and sequential enzyme hydrolysis, Allow indicated a coarse face.
(TIF)

Table S1 **Variation of two-forms of lignin in** ***Miscanthus*** **accessions (n = 79).**
(DOCX)
