# Supplementary figures and images for: The Minor Wall-Networks between Monolignols and Interlinked-Phenolics Predominantly Affect Biomass Enzymatic Digestibility in Miscanthus
Source: PLoS One. 2014 Aug 18;9(8):e105115. doi: 10.1371/journal.pone.0105115 (PMC4136839; doi:10.1371/journal.pone.0105115)

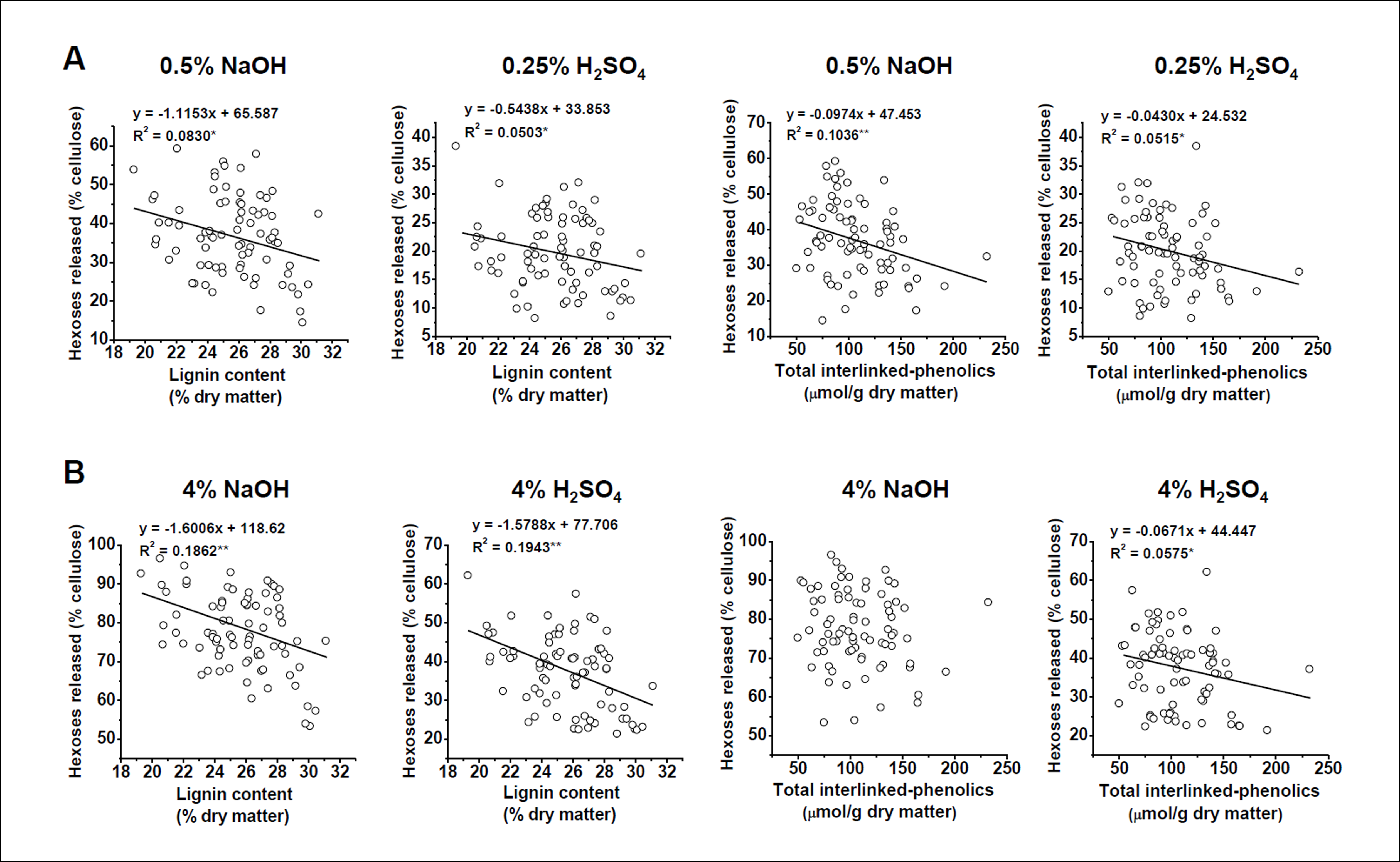

Supplement: Figure S1 — Correlation of lignin and interlinked-phenolics with hexoses yield released from enzymatic hydrolysis. (A) Pretreated with 0.5% NaOH and 0.25% H2SO4; (B) Pretreated with 4% NaOH and 4% H2SO4 in Miscanthus accessions (n = 79). * and ** Indicated the significant correlation coefficient values at p<0.05 and 0.01, respectively. (TIF) [file pone.0105115.s001.tif]

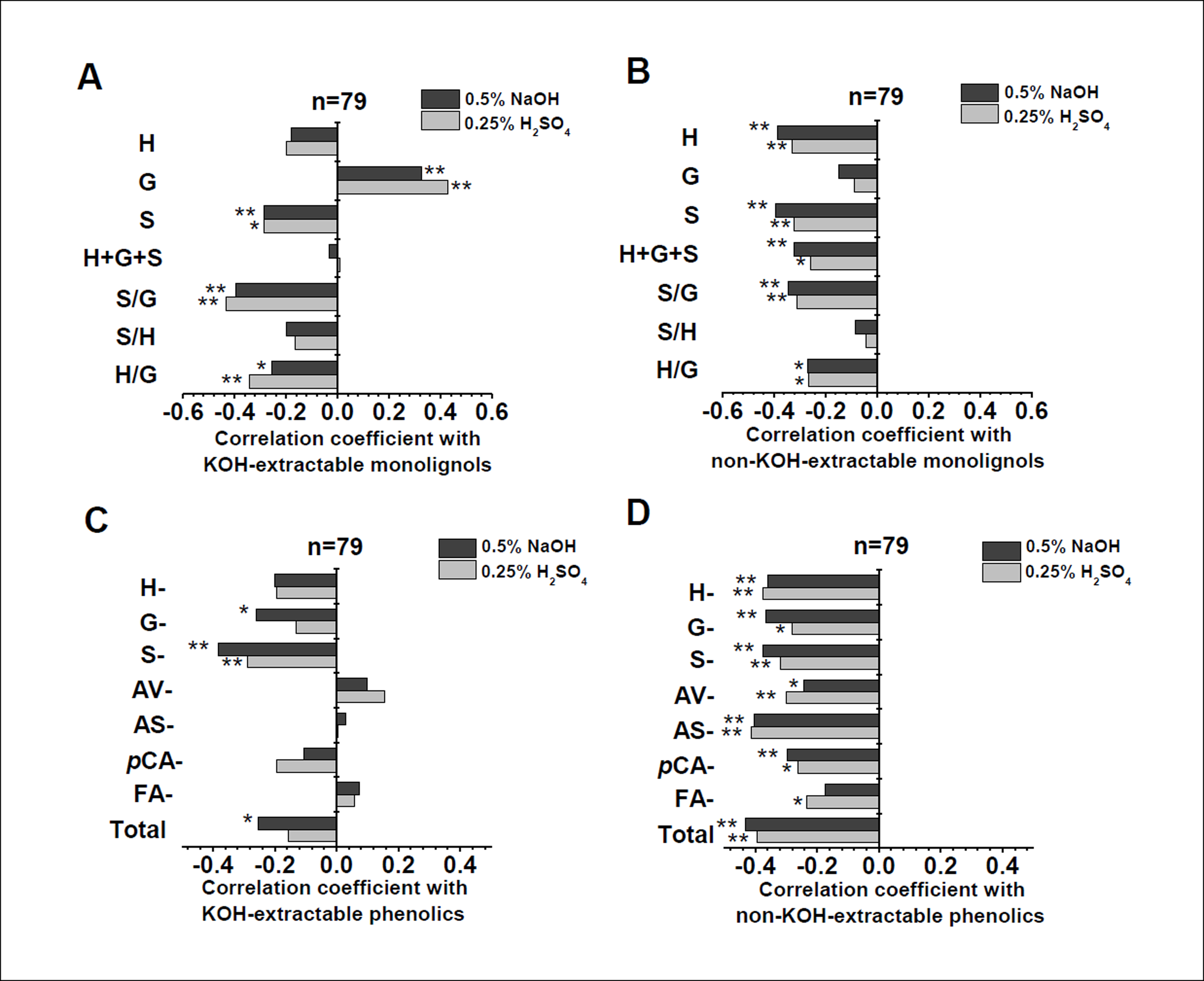

Supplement: Figure S2 — Correlation of monolignols and interlinked-phenolics with hexoses yield released from enzymatic hydrolysis under various pretreatments. (A) KOH-extractable monolignols; (B) Non-KOH-extractable monolignols; (C) KOH-extractable phenolics; (D) Non-KOH-extractable phenolics. * and ** Indicated the significant correlation coefficient values at p<0.05 and 0.01, respectively (n = 79). (TIF) [file pone.0105115.s002.tif]

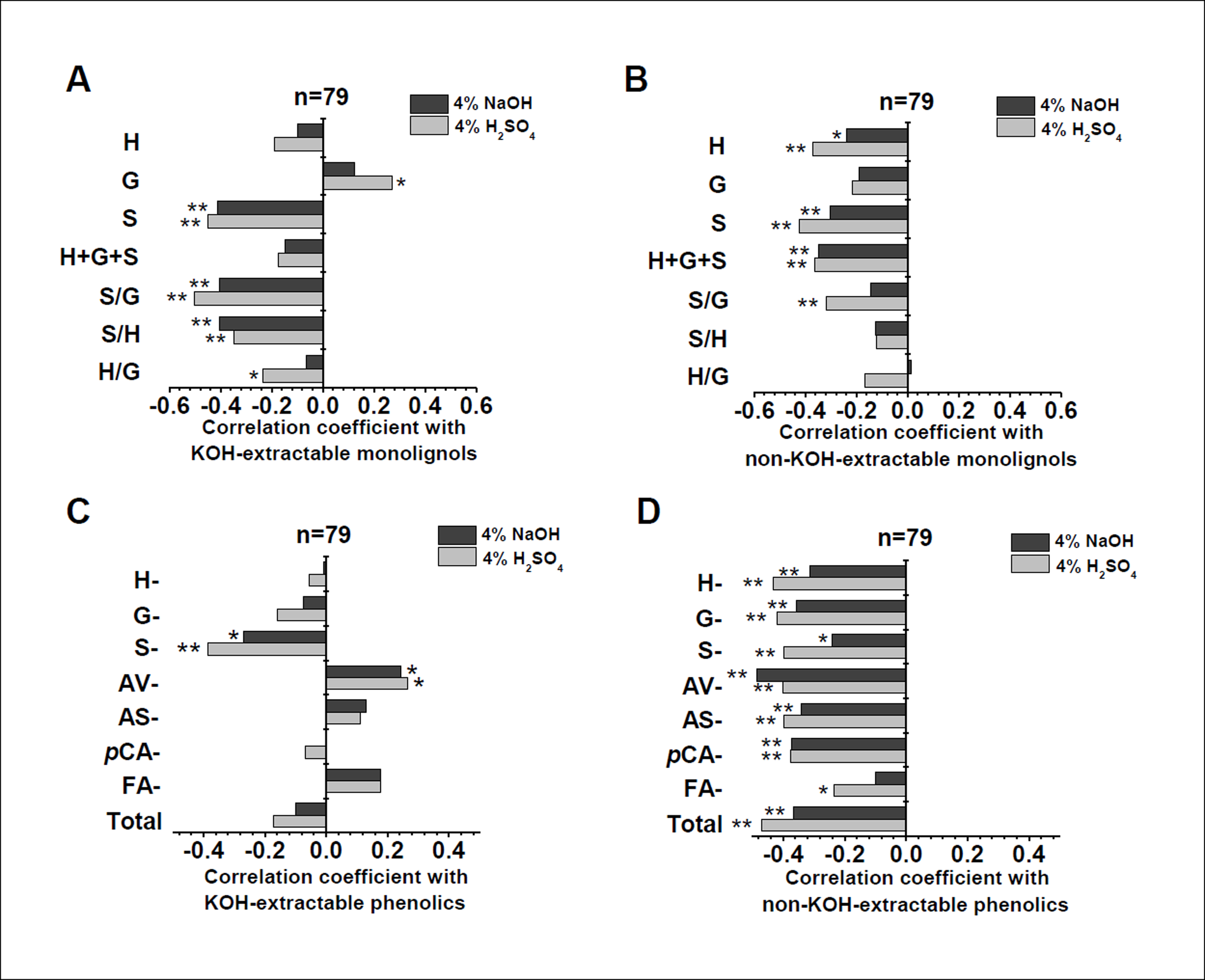

Supplement: Figure S3 — Correlation of monolignols and interlinked-phenolics with hexoses yield released from enzymatic hydrolysis under various pretreatments. (A) KOH-extractable monolignols; (B) Non-KOH-extractable monolignols; (C) KOH-extractable phenolics; (D) Non-KOH-extractable phenolics. * and ** Indicated the significant correlation coefficient values at p<0.05 and 0.01, respectively (n = 79). (TIF) [file pone.0105115.s003.tif]

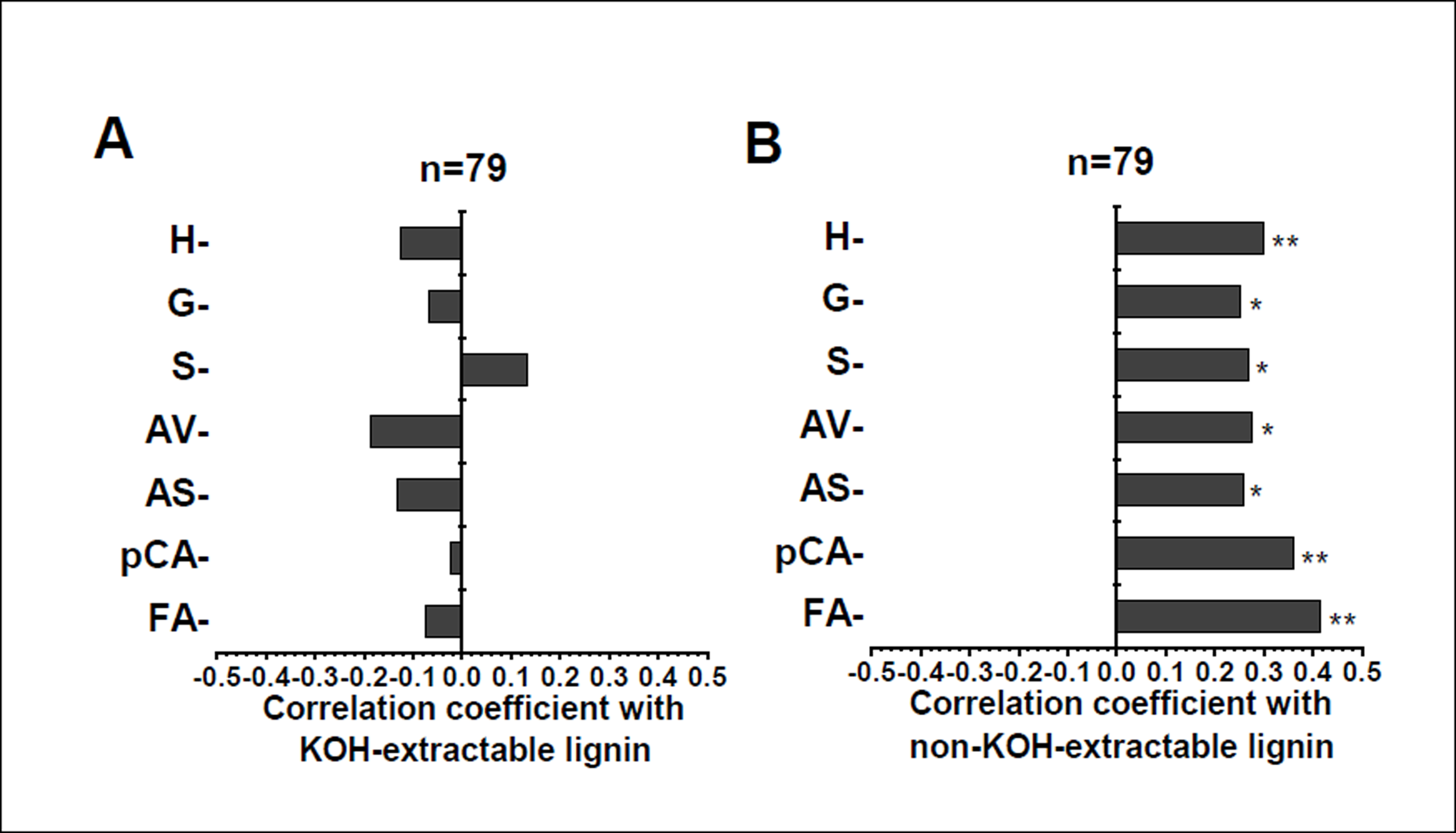

Supplement: Figure S4 — Correlation between seven interlinked-phenolics and lignin in Miscanthus . (A) KOH-extractable lignin; (B) Non-KOH-extractable lignin. * and ** Indicated the significant correlation coefficient values at p<0.05 and 0.01, respectively (n = 79). (TIF) [file pone.0105115.s004.tif]

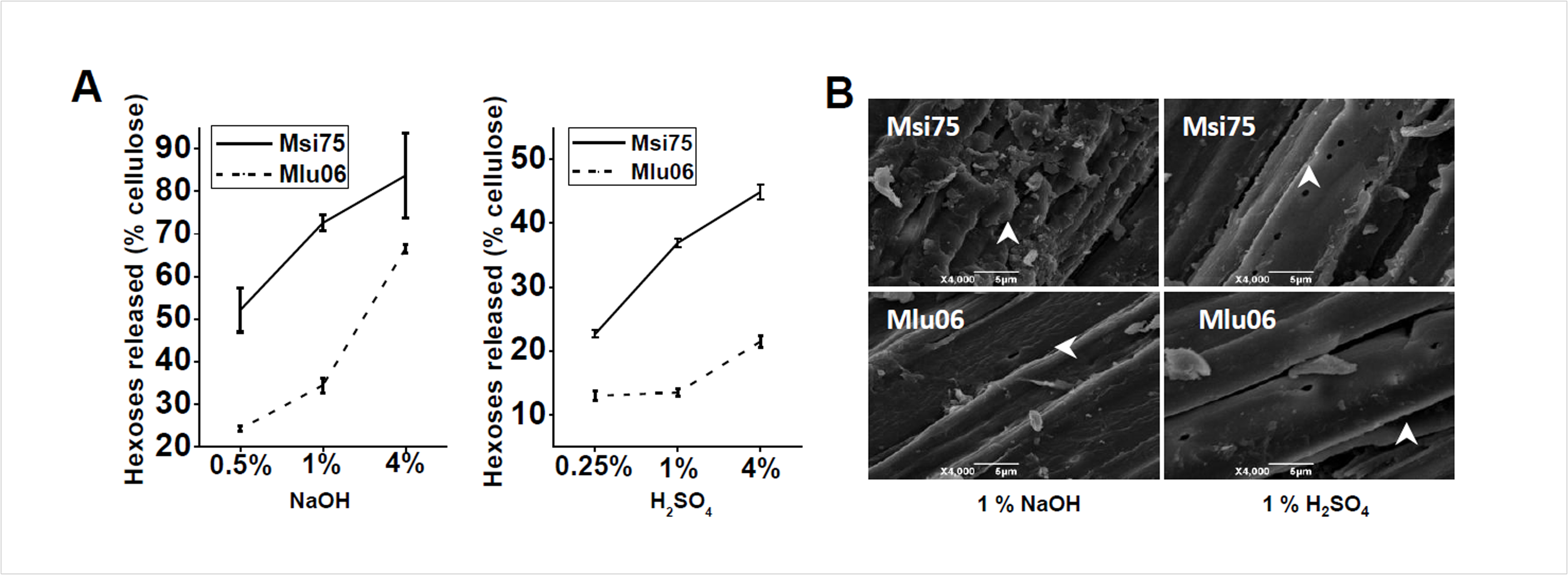

Supplement: Figure S5 — Biomass enzymatic digestibility and scanning electron microscopic observation in representative Miscanthus accessions. (A) Hexoses yields (% cellulose) released from enzymatic hydrolysis after pretreatments of NaOH and H2SO4 at three concentrations as means±SD (n = 3); (B) SEM imagines of the biomass residues obtained from pretreatments of 1% NaOH and 1% H2SO4 and sequential enzyme hydrolysis, Allow indicated a coarse face. (TIF) [file pone.0105115.s005.tif]
