# Supplementary material for: The Minor Wall-Networks between Monolignols and Interlinked-Phenolics Predominantly Affect Biomass Enzymatic Digestibility in Miscanthus
Source: PLoS One. 2014 Aug 18;9(8):e105115. doi: 10.1371/journal.pone.0105115 (PMC4136839; doi:10.1371/journal.pone.0105115)
Supplement: Table S1 — Variation of two-forms of lignin in Miscanthus accessions (n = 79). (DOCX) [file pone.0105115.s006.docx]

**Table S1 Variation of two-forms of lignin in *Miscanthus* accessions (n=79).**

|  |  | Monolignol composition (μmol/g dry matter) | | | |
| --- | --- | --- | --- | --- | --- |
|  |  | H | G | S | Total |
| *Miscanthus* (n=79) | KOH-  extractable | 347.86 (26.70%)* | 628.37 (48.23%) | 326.55 (25.07%) | 1302.79 (80.03%) |
|  |  | (227.03~570.56)# | (387.63~867.75) | (94.59~551.53) | (831.47~1582.4) |
|  |  |  |  |  |  |
|  | Non-KOH-  extractable | 45.38 (13.96%) | 163.25 (50.22%) | 116.46 (35.83%) | 325.08 (19.97%) |
|  |  | (6.00~119.18) | (25.27~284.39) | (15.08~228.81) | (46.35~618.25) |
|  |  |  |  |  |  |
|  | Total | 393.24 (24.16%) | 791.62 (48.63%) | 443.01 (27.21%) | 1627.87 (100%) |
|  |  | (277.31~621.73) | (509.25~1126.92) | (261.44~708.88) | (1060.35~2457.53) |

H: *p*-Hydroxyphenyl units, G: Guaiacyl units, S: Syringyl units. ***** Mean values and percentage, **#** Minimum and maximum values.
